# Neuro-cryptococcosis in an immunocompetent individual with radiologically atypical findings: a case report and review of literature

**DOI:** 10.1093/omcr/omad016

**Published:** 2023-03-25

**Authors:** Binit U Regmi, Bishnu D Pathak, Ram C Subedi, Bishal Dhakal, Suhail Sapkota, Sushil Joshi, Ujjawal Poudel, Raju Poudel

**Affiliations:** Department of Neurology, Jibjibe Primary Health Care Center, Rasuwa, Nepal; Department of Neurology, Jibjibe Primary Health Care Center, Rasuwa, Nepal; Department of Neurology, Grande International Hospital, Kathmandu, Nepal; Department of Neurology, Nepalese Army Institute of Health and Sciences, Kathmandu, Nepal; Department of Neurology, Nepalese Army Institute of Health and Sciences, Kathmandu, Nepal; Department of Neurology, Dodhara Primary Health Care Center, Mahendranagar, Nepal; Department of Neurology, Jibjibe Primary Health Care Center, Rasuwa, Nepal; Department of Neurology, Nepalese Army Institute of Health and Sciences, Kathmandu, Nepal

## Abstract

We present a case of a 29-year-old immunocompetent female without any known comorbidities with intermittent headache and vomiting who was ultimately diagnosed with cryptococcal meningitis (CM). Though her neuroimaging findings were atypical to those commonly found in CM, she was diagnosed with CM with a cryptococcal antigen test. However, in contrast to the good prognosis as stated in the literature, she died during her course stay at the hospital. Therefore, cryptococcosis should be taken as differentials, even in an immunocompetent individual presenting with features suggestive of meningitis, to prevent the worst clinical outcome.

## INTRODUCTION

Cryptococcal meningitis (CM) is a brain infection caused by the fungus Cryptococcus (either *Cryptococcus neoformans or Cryptococcus gattii*). It is one of the most typical causes of meningitis in immunocompromised people, particularly those with Human Immuno-deficiency Virus/Acquired Immune Deficiency Syndrome (HIV/AIDS) [1]. Although antiretroviral drugs reduce the prevalence of CM in HIV patients, the trend in an immunocompetent host is assumed to rise over the years [2]. Annually, there are 223 100 cases of CM worldwide with the largest burden in sub-Saharan Africa, South and South-East Asian regions [1, 3].

Common meningeal symptoms, such as headache and neck stiffness, are usually present in classic CM. However, other systemic signs of disseminated illness, such as pneumonia and dermatological symptoms, may also be present [1]. Therefore, evaluation of immunocompetent patients with subacute to chronic meningitis for *C. neoformans* is needed. Even though serum cryptococcal antigen and radiographic imaging help identify CM, lumbar puncture is necessary for a conclusive diagnosis [1, 4]. The lumbar puncture with elevated opening pressure, predominantly mononuclear cells, demonstration of encapsulated yeast with India ink and positive cerebrospinal fluid (CSF) culture of the organism are typical findings confirming the diagnosis [4].

The examination for CM is infrequently performed in low-income countries like Nepal among immunocompetent individuals who exhibit meningitis-like symptoms since the diagnosis is frequently overlooked. Unfortunately, such an immunocompetent patient with CM carries a higher mortality rate than those with an immunosuppressed state [5]. We present a case of a 29-year-old immunocompetent female who experienced chronic blurred vision, headaches and vomiting for 3 months before being diagnosed with CM.

## CASE REPORT

A 29-year-old married female presented to our center with complaints of intermittent headaches followed by vomiting for the last 3 months. Each episode of headache was acute in onset, located in the parieto-occipital region, moderate in intensity and associated with vomiting most of the time. The pain was slightly relieved with analgesics from the local pharmacy. But, for the last 20 days, the patient reported an increase in the frequency and severity of headaches and multiple vomiting episodes. She also had a fever but the temperature was not recorded. There was no history of trauma, chronic intake of medications, loss of consciousness, abnormal body movements and recent change in behaviors. There was no significant medical or surgical history suggestive of immunodeficiency. She was a reformed smoker and occasionally consumed alcohol.

At presentation, the patient was conscious and well oriented to time, place and person with a Glasgow Coma Score of 15 out of 15. The sensory, motor, cranial nerve and cerebellar examination did not reveal any abnormal findings. The meningeal signs like neck stiffness and Kernig’s sign were absent. Direct ophthalmoscopic examination showed no evidence of optic disk swelling or papilledema. Vital signs were within normal limits (blood pressure: 110/80 mmHg; respiratory rate: 18 breaths per minute and temperature: 98.6 degrees Fahrenheit) except for tachycardia (110 bpm). The systemic examination was unremarkable. Baseline investigations were within the reference range. The serum glucose measured was 90 mg/dl. The patient was admitted for further evaluation.

**Figure 1:**
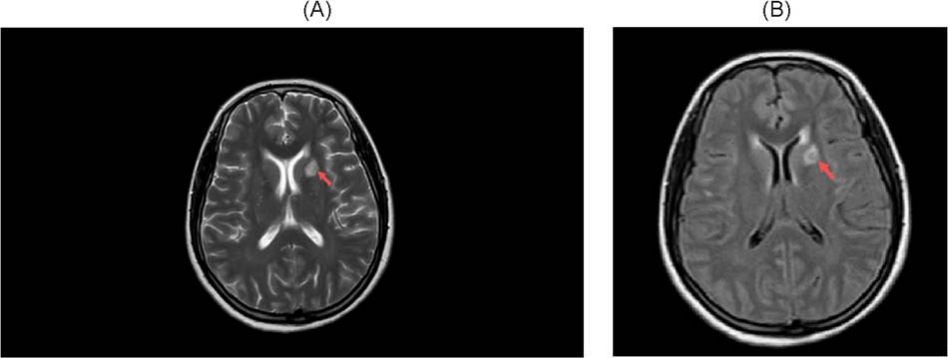
MRI of head. (**a**) T2-weighted view. (**b**) FLAIR view. Red arrows showing ring-like lesion in the left lentiform nucleus.

**Figure 2:**
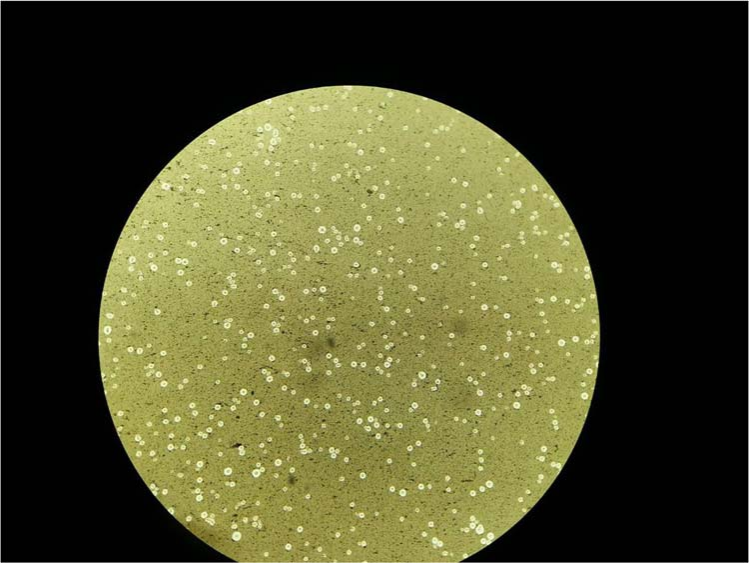
India ink staining of CSF showing *C. neoformans.*

The magnetic resonance imaging (MRI) showed a ring-like lesion in the left lentiform nucleus with an eccentric nodule and surrounding edema. These features were suggestive of neurocysticercosis (colloidal vesicular stage; Fig. 1). Lumbar puncture for CSF analysis was done on the next day which revealed normal white cell count (less than five cells), high protein (50 mg/dl; 15–45 mg/dl), low glucose (30 mg/dl; 50–80 mg/dl) and normal adenosine deaminase (8 U/L; < 10 U/L) levels. The opening pressure was 12 cm of H_2_O. Gram staining showed multiple budding yeast cells resembling *Cryptococcus* sp. Likewise, India ink preparation of the CSF sample showed a capsule structure resembling *C. neoformans* (Fig. 2). The diagnosis of CM was made a week after the admission. A part of the sample was sent outside for a cryptococcal antigen test as it was not available in our center.

The patient was transferred to intensive care unit (ICU), and central venous and arterial access was obtained. Parenteral amphotericin B (liposomal; 3 mg/kg/day) was started for CM. Likewise, oral fluconazole (800 mg per day) was also administered as flucytosine was not available in our setting. Therapeutic CSF drainage was planned in case of persistent headache and/or vomiting. Other supportive treatment measures including chest physiotherapy and mobilization were provided. The following day of ICU admission, she developed metabolic acidosis with respiratory compensation, which was managed conservatively. She developed hypotension (90/60 mmHg), tachycardia (112 bpm) and tachypnea (26 breaths per minute). The serum lactate level was elevated (4.5 mmol/L), and the diagnosis of septic shock was made. Noradrenaline (10 mcg/min) was started for the same. Parenteral meropenem (1 g every 8 h) was added for worsening oxygenation with the differential diagnosis of hospital-acquired pneumonia or aspiration pneumonia. The echocardiography showed normal left ventricular function with an ejection fraction of 50–55%, severe tricuspid regurgitation with estimated pulmonary artery systolic pressure of 60 mm Hg and severely dilated right cardiac chambers. Therefore, furosemide (80 mg IV) was administered for volume overload.

In the meantime, the antigen test for *Cryptococcus* came back positive. The septic shock resolved gradually, and the noradrenaline infusion was stopped. Therapeutic lumbar puncture was done for 2 days and 23 mL of CSF was removed as the opening pressure was high (>35 cm of H_2_O; 10–20 cm of H_2_O). The rest of the intensive care and monitoring was continued. But, after 2 days of slight improvement (clinically), the condition of the patient suddenly deteriorated, and ultimately, she passed away on the 7th day of her ICU stay. The actual cause of the death could not be determined as the postmortem examination was not conducted.

## DISCUSSION

In contrast to Cryptococcosis that is considered a classical disease with certain underlying diseases pathology or predisposing factors, cryptococcosis in phenotypically normal individuals is on the rise [5]. The global burden of CM in the immunocompromised population, particularly in the HIV-AIDS population, has been extended with an estimated prevalence of 6.0% [6]. A comprehensive report from 2015 estimates the total burden of CM (in HIV/AIDS and immunocompromised patients) to be 164 (0.6 per 100 000) in Nepal [7]. The occurrence of CM in immunocompetent individuals (non-HIV and non-transplant patients) has been increasing steadily with most centers reporting up to 20% of cases in the non-immunocompromised population [5]. According to a study by Hevey *et al*. [2], the 90-day mortality was higher in immunocompetent groups (41.1%) as compared with immunocompromised groups (15.2% in HIV and 12.2% in transplant groups).

Two fungi are implicated in invasive cryptococcosis, *C. neoformans* and *C. gatti*, both encapsulated yeasts. However, *C. neoformans* has been associated with the immunocompromised host in contrast to *C. gatti*, which is shown to occur with immunocompetent hosts [5, 8]. Cryptococcus species have a propensity for the central nervous system, particularly in immunocompromised patients, because of their multiple virulence factors. However, CM in immunocompetent patients is difficult to explain. The possible explanations could be because of exposure to cryptococcal strain with high pathogenicity, high level of pathogen exposure, or subtle or undetectable immune deficits. Such deficits could be because of alcoholism, diabetes mellitus or autoimmune conditions [8]. Our case did not have any of those subtle causes for immune deficits, because of which we labeled our case as apparently immunocompetent.

The Cryptococcus species, in immunocompetent individuals, have more propensity for developing pulmonary cryptococcosis than in immunocompromised individuals. Similarly, the neuroimaging findings are more localized and include hydrocephalus in immunocompetent patients [5, 8]. The clinical picture of CM in immunocompetent patients tends to have typical features including fever, stiff neck, headache, vomiting or altered mental status in contrast to immunocompromised individuals, who have only subtle clinical features [8]. Regarding our case, she had neurological involvement with fever and vomiting as the initial presentation and later neurological deterioration. The neuroimaging findings did not show hydrocephalus, instead had a ring-enhancing lesion resembling findings of the colloidal vesicular stage of neurocysticercosis. However, there can be findings in imaging like cerebral infarction, granulomas and pseudocysts [5, 8].

As for Nepal, in two case reports of CM in immunocompetent patients, both had apparently immunocompetent patients without any subtle immune deficits. And with the appropriate treatment as per recommended guidelines [1, 8], both had good prognoses [9, 10]. However, it was quite the opposite regarding our patient, who died during the course of treatment in the ICU of our center. This could be possibly because of neurological deterioration secondary to raised intracranial pressure. There is conflicting evidence on the prognosis of CM in both immunocompetent and immunocompromised patients, with some favoring immunocompetent individuals [5, 9, 10]. However, mortality can also be pertinent in immunocompetent individuals because of the unavailability of investigative tools, frequent overlooking of the diagnosis and delayed presentation.

## CONCLUSION

Hence, cryptococcosis should be considered despite an apparent immunocompetent individual without any known comorbid conditions presenting with features suggestive of meningitis. The early diagnosis and appropriate treatment of CM could lead to a better neurological outcome and prognosis for the patient.

## CONFLICT OF INTEREST STATEMENT

None declared.

## ETHICAL APPROVAL

Not applicable.

## CONSENT

A written informed consent was obtained from the patient for the case study.

## GUARANTOR

Bishal Dhakal, Nepalese Army Institute of Health and Sciences, 44 600, Kathmandu, Nepal. Email: swarnimdhakal@gmail.com
